# Supplementary material for: Organization at criticality enables processing of time‐varying signals by receptor networks
Source: Mol Syst Biol. 2020 Feb 24;16(2):e8870. doi: 10.15252/msb.20198870 (PMC7036718; doi:10.15252/msb.20198870)
Supplement: Supplementary file 1 — Expanded View Figures PDF [file MSB-16-e8870-s001.pdf]

Expanded View Figures

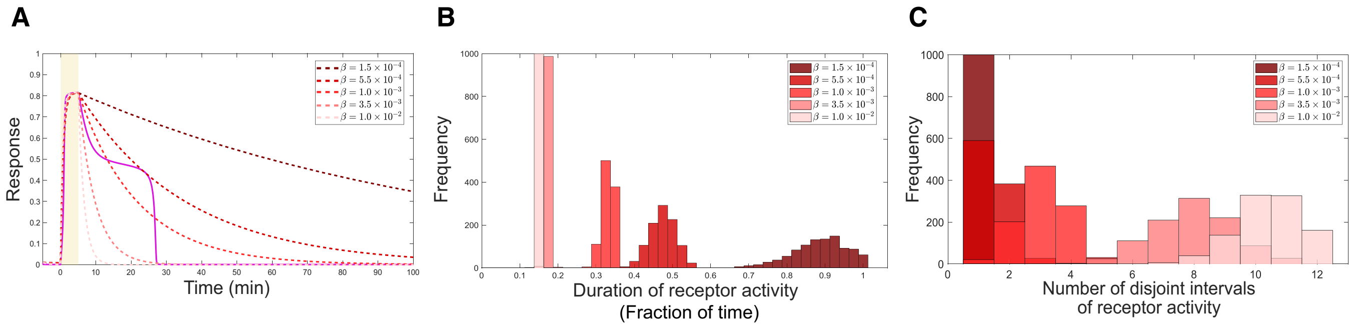

Figure EV1. Information processing features for an exponentially decaying relaxation process.

A Temporal receptor activity profiles for distinct decay rates β (dashed red lines; model and respective parameters in Materials and Methods) as compared to the temporal profile for organization at criticality (magenta, equivalent to Fig 2C). Yellow shaded region: growth factor pulse duration.

B Corresponding distributions of total duration of receptor activity (as a fraction of total time, 480 min) calculated for growth factor train pulses constructed as in Fig 3B.

C Distributions of number of disjoint intervals of receptor activity for the different β values. The distributions are estimated from 1,000 different realizations of growth factor pulse trains as in Fig 3.

© 2020 The Authors
