# Supplementary material for: Organization at criticality enables processing of time‐varying signals by receptor networks
Source: Mol Syst Biol. 2020 Feb 24;16(2):e8870. doi: 10.15252/msb.20198870 (PMC7036718; doi:10.15252/msb.20198870)
Supplement: Supplementary file 2 — Movie EV1 [file MSB-16-e8870-s002.zip › Movie_EV1.pdf]

Movie EV1. **Transient memory in receptor activity via a "ghost" attractor.** Phase space transitions and a trajectory (left) depicting receptor responsiveness (right, green – receptor activity, grey – fraction of ligand-bound receptors) to single stimulus pulse (yellow) for positioning at criticality. Orange circles - stable steady states, blue circle - unstable steady state, dashed line - separatrix. The results depict stochastic realization of the model equations (1) including ligand binding dynamics (see Materials and Methods 4.1).
