# Supplementary material for: Organization at criticality enables processing of time‐varying signals by receptor networks
Source: Mol Syst Biol. 2020 Feb 24;16(2):e8870. doi: 10.15252/msb.20198870 (PMC7036718; doi:10.15252/msb.20198870)
Supplement: Supplementary file 3 — Movie EV2 [file MSB-16-e8870-s003.zip › Movie_EV2.pdf]

Movie EV2. **Permanent memory in receptor activity with bistable organization.** Single molecule reaction-diffusion simulations (left) of the interaction between receptor, R (red) and P_{DNF} (blue, omitted), for positioning at bistability ($\tilde{\gamma}_{DNF} = 4.55/(\sigma^2\pi)$), using initial conditions with fully active receptor. Active receptor molecules are depicted with dark red, while inactive with light red. The respective fractions of active molecules in time is shown on the right.
