# Supplementary material for: Organization at criticality enables processing of time‐varying signals by receptor networks
Source: Mol Syst Biol. 2020 Feb 24;16(2):e8870. doi: 10.15252/msb.20198870 (PMC7036718; doi:10.15252/msb.20198870)
Supplement: Supplementary file 4 — Movie EV3 [file MSB-16-e8870-s004.zip › Movie_EV3.pdf]

Movie EV3. **Transient memory in receptor activity with critical organization.** Same description as in Movie EV2, for $\tilde{\gamma}_{DNF} = 4.95/(\sigma^2\pi)$, using initial conditions with fully active receptor.
