# Supplementary material for: Organization at criticality enables processing of time‐varying signals by receptor networks
Source: Mol Syst Biol. 2020 Feb 24;16(2):e8870. doi: 10.15252/msb.20198870 (PMC7036718; doi:10.15252/msb.20198870)
Supplement: Supplementary file 5 — Movie EV4 [file MSB-16-e8870-s005.zip › Movie_EV4.pdf]

Movie EV4. **Transient memory in receptor activity with critical organization.** Same as in Movie EV3, with local values of $\tilde{\gamma}_{DNF} P_{DNF,T} / R_T$ color-coded and underlaid. Colors are normalized according to the SN_1 and SN_2 values from the bifurcation diagram in Fig.4B: Red translates to local $R_0 > 1$.
