# Supplementary material for: Organization at criticality enables processing of time‐varying signals by receptor networks
Source: Mol Syst Biol. 2020 Feb 24;16(2):e8870. doi: 10.15252/msb.20198870 (PMC7036718; doi:10.15252/msb.20198870)
Supplement: Supplementary file 6 — Movie EV5 [file MSB-16-e8870-s006.zip › Movie_EV5.pdf]

Movie EV5. **Rapid relaxation of receptor activity to basal levels with monostable organization.** Same description as in Movie EV2, for $\tilde{\gamma}_{DNF} = 6.0/(\sigma^2\pi)$, using initial conditions with fully active receptor.
